# Machine Learning and Wavelet Transform: A Hybrid Approach to Predicting Ammonia Levels in Poultry Farms

**DOI:** 10.3390/ani14202951

**Published:** 2024-10-14

**Authors:** Erdem Küçüktopçu, Bilal Cemek, Halis Simsek

**Affiliations:** 1 Department of Agricultural Structures and Irrigation, Ondokuz Mayıs University, Samsun 55139, Türkiye; bcemek@omu.edu.tr; 2 Department of Agricultural and Biological Engineering, Purdue University, West Lafayette, IN 47907, USA; simsek@purdue.edu

**Keywords:** ammonia, machine learning, wavelet transform, broiler

## Abstract

**Simple Summary:**

With rapid technological advances, the use of machine learning in the poultry sector has increased significantly. The estimation of ammonia concentration with machine learning can greatly impact environmental protection as well as human and animal health. In this paper, an innovative hybrid approach combining machine learning with wavelet transform for ammonia estimation in poultry houses is presented. The results of the study show that these hybrid models are very promising for accurate and efficient ammonia estimation.

**Abstract:**

Ammonia (NH_3_) is a major pollutant in poultry farms, negatively impacting bird health and welfare. High NH_3_ levels can cause poor weight gain, inefficient feed conversion, reduced viability, and financial losses in the poultry industry. Therefore, accurate estimation of NH_3_ concentration is crucial for environmental protection and human and animal health. Three widely used machine learning (ML) algorithms—extreme learning machine (ELM), k-nearest neighbor (KNN), and random forest (RF)—were initially used as base algorithms. The wavelet transform (WT) with ten levels of decomposition was then applied as a preprocessing method. Three statistical metrics, including the mean absolute error (*MAE*) and the correlation coefficient (*R*), were used to evaluate the predictive accuracies of algorithms. The results indicate that the RF algorithms perform robustly individually and in combination with the WT. The RF-WT algorithm performed best using the air temperature, relative humidity, and air velocity inputs with a *MAE* of 0.548 ppm and an *R* of 0.976 for the testing dataset. In summary, applying WT to the inputs significantly improved the predictive power of the ML algorithms, especially for inputs that initially had a low correlation with the NH_3_ values.

## 1. Introduction

Ammonia (NH_3_) can have a significant global impact if it is in the atmosphere [1]. It reacts with other pollutants such as sulfur dioxide (SO_2_) and nitrogen oxides (NO_x_) to form particulate matter (PM_2.5_) [2]. These particles can penetrate deep into the respiratory tract and cause serious health problems such as asthma, bronchitis, cardiovascular disease, and even premature death [3]. In addition, atmospheric NH_3_ contributes to the formation of acid rain, which leads to acidification and eutrophication of soils and rivers [4]. Numerous studies have documented the harmful effects of NH_3_ on the environment, ecosystems, and human health [5,6].

Confined animal feeding operations are primary sources of NH_3_ and pose a significant risk to the health and welfare of animals and their caretakers [6]. NH_3_ is a particularly harmful gaseous pollutant commonly found in poultry farms. Elevated NH_3_ concentrations (e.g., >25 ppm) can lead to reduced weight gain, poor feed conversion, and reduced bird viability, ultimately resulting in financial losses for the poultry industry [7,8]. Therefore, monitoring and assessing ammonia levels in poultry houses is essential.

The concentration of NH_3_ is influenced by various factors, such as the physical properties of the litter (e.g., moisture content, pH, and surface temperature) and the characteristics of the air (e.g., air velocity, relative humidity, and temperature). Although various devices and instruments can monitor NH_3_ concentrations in poultry houses, data collection in large production facilities is challenging. This process is typically labor-intensive, time-consuming, expensive, prone to human error, and requires ongoing calibration [9].

One method for predicting NH_3_ concentration in poultry houses involves determining the input-output relationship between relevant variables based on field measurements. The advantage of ML lies in its ability to efficiently process large datasets, automatically detect patterns, and make predictions based on complex, multi-dimensional input variables. ML algorithms can also be continuously improved with more data, making them highly adaptable to changing conditions in production environments. However, a significant limitation of these ML algorithms is their reliance on data that is not stationary [10].

The wavelet transform (WT) offers an efficient solution to this problem. The WT decomposes a signal into components corresponding to different frequency bands. This decomposition is achieved by applying wavelets, which are mathematical functions that effectively capture both time and frequency information [11,12]. Applying the WT to NH_3_ data can enable improved accuracy in estimation processes.

A literature review shows that a few researchers have recently developed NH_3_ prediction models for agricultural environments using ML algorithms [13]. However, no study has yet performed hybridization (ML-WT) of NH_3_ models for poultry houses, so knowledge on this topic is incomplete and fragmented.

Therefore, this study aims to modify and develop a novel hybrid model to predict NH_3_, combining ML algorithms, specifically extreme learning machine (ELM), k-nearest neighbor (KNN), and random forest (RF), with the WT algorithm for NH_3_ modeling.

## 2. Materials and Methods

### 2.1. Study Area and Measurements

This study was conducted in a poultry house in Samsun, Türkiye (41°70′ N, 36°30′ E). Measurements were taken at the beginning (7th day), middle (21st day) and end (40th day) of eight rearing periods in 2018 and 2019.

A sensor (Guangzhou, China) was used to measure the NH_3_ concentration. The measurements of relative humidity (RH) and temperature (T) were performed with a thermo-hygrometer (Lenzkirch, Germany). Additionally, air velocity (V) distribution was determined by employing a hot-wire anemometer (Tampa, FL, USA). To assess the moisture content of the litter (LMC), the samples were dried in an oven set at 65 °C for 48 h and subsequently weighed in aluminum trays using an analytical balance (Istanbul, Türkiye). The pH of the litter (LPH) was gauged utilizing a pH meter (Sarasota, FL, USA) in a 1:10 solution with distilled water. Additionally, the surface temperature of the litter (LT) was determined by employing a thermal imaging camera (Lenzkirch, Germany). The instruments used in this study, along with their specifications, are listed in Table 1. The measurements were taken at eighty points on the building, simultaneously in three areas, beginning from the front and repeating from the back. Each point was measured three times at 10 s intervals and the average values were recorded.

**Figure 1:**
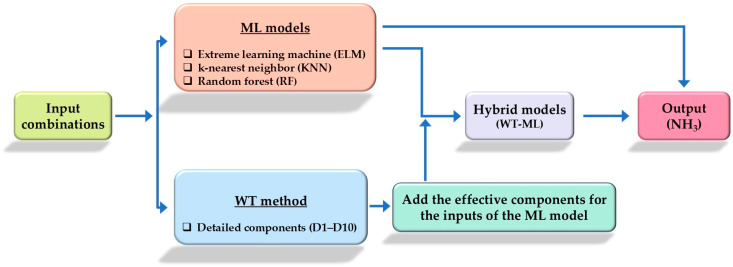
Flowchart of the NH_3_ estimation model.

In this study, three ML algorithms—ELM, KNN, and RF—were chosen as base models for predicting NH_3_ levels. To enhance prediction accuracy, the WT technique was integrated with these algorithms (Figure 1). After applying the wavelet analysis to the original data, the transformed data were used as input for the ML algorithms. This integration resulted in hybrid WT-ML models where the input data were denoised by the WT process, improving model performance.

Model selection was based on a training dataset consisting of 80% of the total data (*n* = 1280) and a testing dataset comprising the remaining 20% (*n* = 320) for the prediction of NH_3_ values. Subsequently, 90% of the training pool was set as the primary training dataset and hyperparameter tuning was performed on 90% of this randomly selected subset. This process was repeated 10 times to ensure the robustness and reliability of the evaluation [14]. Before training the models, all data were standardized to a range between 0 and 1. The analysis was conducted on a PC with a 64-bit Windows 11 operating system, featuring an AMD Ryzen 7 CPU operating at 3.2 GHz and equipped with 16 GB of RAM.

### 2.2. Machine Learning (ML) Algorithms

The KNN is a fundamental ML algorithm known for its simplicity and effectiveness. Its importance lies in its versatility in various areas, including classification, regression, and anomaly detection. One of its primary advantages is its straightforward implementation and intuitive concept: it classifies a data point by a majority vote of its k nearest neighbors, where “k” is a user-defined parameter. KNN is ideal for scenarios where the decision boundary is irregular or mathematically challenging. However, like any method, it has its limitations. Computational complexity increases with the dataset’s size, making it less efficient for large-scale applications. In addition, KNN is sensitive to the choice of distance metric and the value of “k”, which can significantly affect its performance. It also has problems with high-dimensional data due to the curse of dimensionality, where the distances between points become less significant as the number of dimensions increases. Despite these limitations, KNN remains a valuable tool in the ML toolbox, especially for smaller datasets or as a baseline for more sophisticated algorithms [15].

The RF is a powerful ensemble learning method that is popular for its robustness and versatility in ML. It is excellent for classification and regression tasks while being resistant to overfitting. By combining the predictions of multiple decision trees trained on different subsets of data, RF reduces variance and improves generalization. A key advantage is its ability to process large datasets with high dimensionality and mixed data types without much pre-processing. In addition, RF provides estimates of feature importance that are helpful in feature selection and model interpretation. However, the main limitations of RF include its lower interpretability compared to simpler models such as decision trees and its high computational cost, especially with large datasets and many trees. Despite these drawbacks, RF is widely preferred due to its strong performance, scalability, and ease of use [16].

The ELM is a relatively novel and efficient learning algorithm that has attracted attention, especially in neural networks, due to its simplicity and effectiveness. Its importance lies in its ability to provide fast and accurate solutions to various ML problems, including classification, regression, and clustering. Unlike traditional neural network training algorithms that require iterative optimization processes, ELM adopts a one-shot learning approach in which the parameters of the hidden layer are randomly initialized and then fixed. This significantly reduces training times, making ELM particularly advantageous for applications with large datasets or real-time processing requirements. In addition, ELM has strong generalization capabilities and often outperforms other methods, especially in scenarios with limited training data. Furthermore, ELM is very flexible and can use different activation functions and kernel types, allowing it to adapt to different problem domains. However, despite its advantages, ELM also has its limitations. One notable drawback is the lack of interpretability, as the random initialization of parameters can obscure the underlying relationships within the data. Furthermore, the performance of ELM can decrease with highly non-linear or complex datasets compared to more sophisticated learning algorithms. Nonetheless, ELM remains a valuable tool in the machine learning toolbox, offering a balance between simplicity, efficiency, and effectiveness for a wide range of applications [17,18].

### 2.3. Linear Regression (LR)

The LR analysis was conducted to determine the linear relation between input variables—LMC, LPH, LT, T, RH, V—and the output variable (NH_3_) [19]. 

### 2.4. Wavelet Transform (WT)

The WT is a mathematical tool used for signal analysis, offering a unique perspective compared to traditional Fourier methods. It decomposes a signal into different frequency components, capturing both time and frequency information simultaneously. Unlike the Fourier transform, which represents signals exclusively in the form of sinusoids, the WT employs wavelets, small wave-like functions that are localized in both the time and frequency domains. This localization allows WT to effectively capture transient features and abrupt changes in signals, making it particularly useful in areas such as image processing, data compression, and noise reduction. In addition, WT offers multi-resolution analysis, enabling the examination of signals at different scales. The adaptability and efficiency of WTs make them a powerful tool for signal processing and analysis in a wide variety of fields [20].

In the WT method, two primary types of transformations exist: the continuous wavelet transform (CWT) and the discrete wavelet transform (DWT) [21]. The DWT offers a significant advantage over the CWT in terms of computation time and data volume. Hence, the classical DWT was selected for this study.

Wavelets preserve both frequency and time domain properties, as defined by the wavelet function (mother wavelet) and the scaling function (father wavelet). The mother wavelet is mathematically expressed as follows:

(1) $$\psi_{a,b}=\frac{1}{\sqrt{a}}\psi \left(\frac{t-b}{a}\right)$$

where *ψ*_*a*,*b*_(*t*) = wavelet function; *a* = frequency or scale (or dilated) parameter; *b* = translation or shifted parameter.

*DWT* can have been calculated as follows for a discrete time-series *x*(*t*) decomposed into several finite subsets, which happens at a discrete-time *t*:

(2) $${DWT}_{m,n}\left(t\right)=2^{-\frac{m}{2}}\int_{-\infty }^{\infty }x\left(t\right)\psi^{*}\left(\frac{t-n2^{m}}{2^{m}}\right)$$

where the wavelet is dilated by “*m*” and shifted by “*n*”; this process allows the wavelet to be tuned and controlled for different frequencies and time shifts.

### 2.5. Model Evaluation

In assessing the predictive accuracy of the algorithms, two commonly employed statistical indices were employed in this study: the correlation coefficient (*R*) and mean absolute error (*MAE*), which were calculated according to the following equations:

(3) $$R=\frac{n\left(\sum XY\right)-\left(\sum X\right)\left(Y\right)}{\sqrt{\left[n\sum X^{2}-{\left(\sum X\right)}^{2}\right]\left[n\sum Y^{2}-{\left(\sum Y\right)}^{2}\right]}}$$

(4) $$MAE=\frac{\sum_{i=1}^{n}\left(\left|X-Y\right|\right)}{n}$$

where *X* is the actual value; *Y* represents the predicted value; *n* denotes is the total amount of data.

## 3. Results

### 3.1. Data Preprocessing

Table 2 summarizes the descriptive statistics for various properties of litter and air for both the training and testing datasets. For LMC, the training dataset ranges from 15.02% to 42.88%, while the testing dataset ranges from 15.75% to 41.59%. The LT values are quite similar, ranging from 20.00 °C to 33.40 °C for the training data and 20.05 °C to 33.00 °C for the testing data. LPH values are consistent across all datasets, a range that varies slightly from 6.02 to 8.34 (training) and 6.05 to 8.26 (testing). The T values range from 19.10 °C to 32.44 °C (training) and 19.10 °C to 31.78 °C (testing), and the RH values range from 50.35 to 79.81% (training) and 50.76 to 79.37% (testing). V values are consistent, with training data ranging from 0.11 to 2.10 m s^−1^ and testing data ranging from 0.12 to 2.05 m s^−1^. The NH_3_ values range from 13.00 to 26.70 ppm in the training data and 13.10 to 26.30 ppm in the testing data.

In this study, different combinations of inputs were employed to estimate NH_3_ concentration, including (i) LMC, (ii) LMC and LPH, (iii) LMC, LPH, and LT, (iv) T, (v) T and RH, (vi) T, RH, and V, and (vii) LMC, LPH, and T.

### 3.2. Evaluation of LR Models’ Performance

Table 3 evaluates the performance of the different LR models in both the training and testing datasets. The LR7 model has the lowest *MAE* with values of 1.402 ppm in training and 1.391 ppm in testing. Conversely, the LR6 model achieves the highest *R* with a training value of 0.829 and a testing value of 0.813. This indicates that both models perform well in predicting NH_3_ levels in poultry farms.

### 3.3. Evaluation of ML Algorithms’ Performance

Three primary hyperparameters need to be adjusted to optimize the KNN algorithm, namely the number of neighbors (k), the leaf size (ls), and the power parameter (p). To optimize the model, a grid search was conducted to identify the best values for these hyperparameters. The parameter ranges tested were 1–10 for ‘p’, 1–30 for ‘ls’, and 1–50 for ‘k’. Table 4 evaluates the performance of the different KNN algorithms with different hyperparameters for both the training and testing datasets. KNN1, which uses only LMC, achieves an *MAE* of 1.559 ppm and an *R* of 0.755 for the testing dataset. KNN2 and KNN3 improve the *MAE* to 1.272 ppm and 1.103 ppm and increase the *R* to 0.807 and 0.832, respectively. KNN4, KNN5, and KNN6, which use different combinations of T, RH, and V, show varying degrees of improvement. Notably, KNN6 (k: 3, ls: 1, p: 1) demonstrates the best performance in the testing dataset with the lowest *MAE* of 0.754 ppm and the highest *R* of 0.933. KNN7, a combination of LMC, PH, and T, also performs well, with a *MAE* of 0.886 ppm and an *R* of 0.901, indicating strong predictive capabilities.

In the RF algorithm, the main hyperparameters are the number of trees (nt), the maximum depth of each tree (d), the minimum number of samples required to split an in-ternal node (ss), and the minimum number of samples needed to reach a leaf node (sl). To optimize the model, a grid search was used to find the best values for these parameters. The hyperparameter ranges considered were 1–10 for ‘sl’, 1–10 for ‘d’, 2–10 for ‘ss’, and 1–50 for ‘nt’. Table 5 compares the performance of the different RF algorithms (RF1 to RF7) with different inputs for predicting the NH_3_ variables. RF1, which uses only LMC as input, shows moderate performance with an *MAE* of 1.544 ppm and an *R* of 0.755 on the testing data. The addition of LPH in RF2 significantly enhances the performance, reducing the *MAE* to 1.181 ppm and increasing the *R* to 0.824. The inclusion of LT in RF3 further improves the results, achieving an *MAE* of 0.975 ppm and an *R* of 0.879. Models RF4, RF5, and RF6 investigate different combinations of T, RH, and V, with RF6 achieving the best performance (*MAE*: 0.644 ppm, *R*: 0.953). Finally, RF7, which combines LMC, LPH, and T, achieves a balanced performance with an *MAE* of 0.819 ppm and an *R* of 0.919, indicating robust predictive capabilities.

In the ELM algorithm, the key hyperparameters are the number of hidden nodes (hn), the activation function (af), and the regularization parameter (rp). The hyperparameter ranges were 1–200 for ‘hn’, [sigmoid, tanh, relu] for ‘af’, and 0.0001-0.1 for ‘rp’. Table 6 presents the performance metrics of the different ELM algorithms with various inputs. ELM6, which uses T, RH, and V as inputs with hyperparameters (180, sigmoid, 0.001), shows the best performance with the lowest *MAE* (1.089 ppm) and the highest *R* (0.893) in the testing phase. In contrast, ELM1, which uses only LMC as input with hyperparameters (160, sigmoid, 0.001), exhibits the least favorable performance metrics.

### 3.4. WT Analysis with ML Algorithms

In the second phase of the study, KNN, RF, and ELM were combined with WT, i.e., WT-KNN, WT-RF, and WT-ELM, respectively. In this phase, ten levels of wavelet decomposition were applied. Table 7 provides the correlation coefficients between the wavelet details and the NH_3_ data series. Of all the components, D6 and D10 have the highest impact on NH_3_ properties.

The process of wavelet decomposition serves as a crucial step in identifying the components that are critical for NH_3_ estimation and facilitates the discrimination and elimination of irrelevant or inactive components from the raw data. In this study, components with strong correlations to the original NH_3_ data series were chosen. Specifically, for LMC, all components with correlations from 0.08 to 0.33 were used; for LPH, components from D3 to D10 (0.11 to 0.32); for LT, components D3 to D9 (−0.09 to 0.31); for T, components from D2 to D10 (−0.06 to 0.32); for RH, all components except D1, D2, and D7 (0.10 to 0.30); and for V, components D3, D4, D5, D6, and D8 (−0.12 to −0.29) were included in forming the new data series. These selected components were then incorporated into ML models to develop the hybrid models, as detailed in Table 8.

### 3.5. Performance Comparison of Different Algorithms

**Figure 2:**
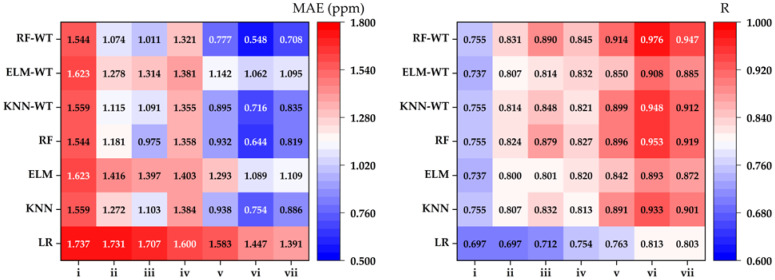
Performance metrics (*MAE* and *R*) of the best algorithms for various input combinations in the test dataset.

The visual comparison in Figure 2 highlights the *MAE* and *R* values for various algorithms across different input combinations within the testing dataset. Notably, the input combination (vi) stands out, as it yields the lowest *MAE* values while achieving the highest *R* values, particularly for the WT algorithms. This suggests that the combination is the most effective. The findings indicate that models incorporating the inputs T, RH, and V produce the most reliable results. Additionally, the results demonstrate that ML-WT algorithms significantly outperform traditional ML algorithms in terms of estimation performance.

The most promising combinations in the ML algorithms (KNN6, RF6, and ELM6) were enhanced with WT, as displayed in Table 9. The performance of the hybrid models (KNN6-WT, RF6-WT, and ELM6-WT) surpassed that of the individual ML algorithms (KNN6, RF6, and ELM6), resulting in a significant reduction in *MAE* values and an increase in *R*. In particular, the RF6-WT model demonstrated an *MAE* reduction of 14.907% and an increase in *R* of 2.994%. Similarly, the KNN6-WT model achieved an *MAE* reduction of 5.040% and an increase in *R* of 1.207%. The ELM6-WT model also performed well, with an *MAE* reduction of 2.479% and an increase in *R* of 1.542% compared to their respective standalone ML algorithms.

## 4. Discussion

With a focus on the importance of model design and input dataset choice, this research evaluates NH_3_ prediction accuracy using both standalone ML models (KNN, RF, ELM) and hybrid ML-WT models in a poultry farm. For a computational model to be effective and precise, the careful selection of input parameters is critical. Analyzing various parameter combinations and assessing their influence on model performance with suitable metrics is necessary. Within the scope of this research, T, RH, and V have been established as the most impactful inputs in NH_3_ estimations.

During the NH_3_ modeling phase, three ML methods—KNN, RF, and ELM—were chosen for their proven effectiveness in various applications as indicated by previous literature [22,23,24,25,26,27,28]. Hyperparameters for each model were determined by a grid search. RF models, when compared to ELM and KNN models, provided more accurate NH_3_ estimates consistently. Some studies on ammonia estimation are available in the literature. For example, the researchers in [13] found that using an integrated adaptive neuro-fuzzy inference system with subtractive clustering (ANFIS-SC) provided NH_3_ concentration predictions with a RMSE of 1.130 ppm and a R^2^ of 0.858. The authors of [29] found that the ML model showed satisfactory predictive performance with RMSE and R^2^ values of 21.15 mg-N.m^−3^ and 0.85, respectively.

In contrast to previous NH_3_ estimation studies, this research incorporates WT into the ML model to improve its predictive capabilities. Although wavelet transform has shown success in various fields [30,31,32,33], its use in poultry farming, particularly for NH_3_ prediction, is not well-explored, emphasizing the originality and innovative aspect of this study. Future studies could investigate the integration of additional ML techniques, such as deep learning models, to further improve the accuracy of NH_3_ prediction. Furthermore, extending the dataset to include different environmental conditions and poultry house configurations could improve the generalizability of the model.

## 5. Conclusions

This study introduces a new hybrid ML-WT modeling approach for estimating NH_3_. Initially, three different ML algorithms—KNN, RF, and ELM—were employed. The WT was then incorporated into these ML algorithms to boost the accuracy of NH_3_ estimation. The key findings of this research are outlined below.

The combination of T, RH, and V has been found to yield the most reliable estimates. However, it is also important to consider scenarios where the properties of the litter itself are relevant. In such cases, a combination of LMC, LPH, and T can be an effective alternative.

Among the selected ML algorithms—KNN, RF, and ELM—the RF6, which combines T, RH, and V, achieves the best performance with an *MAE* of 644 ppm, and an *R* of 0.953.

Compared to the individual ML models, the hybrid models (KNN-WT, RF-WT, and ELM-WT) offer improved accuracy. The RF6-WT model stands out with the most favorable performance for NH_3_ prediction, with an *MAE* of 0.548 ppm, and an *R* of 0.976 in the test dataset.

To conclude, this research advocates for the effectiveness of hybrid ML-WT techniques in accurate NH_3_ estimation and recommends them as a practical alternative.

## Figures and Tables

**Table 1:** List of instruments used in this study and their specifications.

Instrument	Country	Measured Variables	Specification
Smart Sensor, AR8500	China	NH_3_	Resolution: 0.01 ppm Accuracy: ±0.2%
Testo, 605i	Germany	T and RH	Resolution: 0.1 °C and 0.1% RH Accuracy: ±0.5 °C and ±1% RH
PCE, PCE-423	USA	V	Resolution: 0.01 ms^−1^ Accuracy: ±5%
Elektromag, M40P	Türkiye	LMC	Resolution: 1 °C Accuracy: ±1 °C
PCE, PH20S	USA	LPH	Resolution: 0.01 pH Accuracy: ±0.1 pH
Testo, 875–2i	Germany	LT	Resolution: 0.1 °C Accuracy: ±2 °C

**Table 2:** Summary of litter and air parameters in poultry farm.

		LMC (%)	LT (°C)	LPH	T (°C)	RH (%)	V (m s^−1^)	NH_3_ (ppm)
Training	Min	15.02	20.00	6.02	19.10	50.35	0.11	13.00
	Max	42.88	33.40	8.34	32.44	79.81	2.10	26.70
	Mean	30.69	27.87	7.49	24.73	64.66	0.56	19.38
	SD	6.62	2.49	0.54	3.32	6.39	0.54	3.04
	Sk	−0.38	−0.20	−1.30	−0.02	0.25	2.59	0.23
	Kr	−0.93	−0.09	0.65	−0.99	−0.67	7.01	−0.89
Testing	Min	15.75	20.05	6.05	19.10	50.76	0.12	13.10
	Max	41.59	33.00	8.26	31.78	79.37	2.05	26.30
	Mean	30.16	28.02	7.46	24.90	64.59	0.52	19.13
	SD	6.75	2.35	0.54	3.36	6.65	0.46	3.06
	Sk	−0.35	−0.24	−1.22	−0.07	0.33	2.62	0.47
	Kr	−1.02	−0.17	0.44	−0.92	−0.71	7.95	−0.70

Min: Minimum, Max: Maximum, Mean: Mean, SD: Standard deviation, Sk: Skewness, Kr: Kurtosis.

**Table 3:** Evaluation performance metrics for LR models.

Inputs	Models	Training		Testing	
		*MAE*	*R*	*MAE*	*R*
LMC	LR1	1.715	0.729	1.737	0.697
LMC, LPH	LR2	1.702	0.730	1.731	0.697
LMC, LPH, LT	LR3	1.667	0.749	1.707	0.712
T	LR4	1.659	0.753	1.600	0.754
T, RH	LR5	1.626	0.763	1.583	0.763
T, RH, V	LR6	1.412	0.829	1.447	0.813
LMC, LPH, T	LR7	1.402	0.811	1.391	0.803

**Table 4:** Evaluation metrics and hyperparameters for KNN algorithms.

Inputs	Model	Hyperparameters (k, ls, p)	Training		Testing	
			*MAE*	*R*	*MAE*	*R*
LMC	KNN1	19, 11, 1	1.305	0.826	1.559	0.755
LMC, LPH	KNN2	5, 3, 1	0.997	0.892	1.272	0.807
LMC, LPH, LT	KNN3	5, 5, 1	0.847	0.914	1.103	0.832
T	KNN4	11, 3, 1	1.249	0.837	1.384	0.813
T, RH	KNN5	3, 5, 1	0.598	0.955	0.938	0.891
T, RH, V	KNN6	3, 1, 1	0.464	0.972	0.754	0.933
LMC, LPH, T	KNN7	5, 5, 1	0.584	0.960	0.886	0.901

**Table 5:** Evaluation metrics and hyperparameters for RF algorithms.

Inputs	Model	Hyperparameters (nt, d, ss, sl)	Training		Testing	
			*MAE*	*R*	*MAE*	*R*
LMC	RF1	20, 5, 2, 10	1.350	0.820	1.544	0.755
LMC, LPH	RF2	25, 10, 3, 5	0.891	0.915	1.181	0.824
LMC, LPH, LT	RF3	30, 10, 2, 2	0.563	0.965	0.975	0.879
T	RF4	20, 10, 5, 10	1.296	0.827	1.358	0.827
T, RH	RF5	30, 10, 2, 2	0.485	0.975	0.932	0.896
T, RH, V	RF6	25, 10, 2, 3	0.343	0.986	0.644	0.953
LMC, LPH, T	RF7	40, 10, 2, 2	0.445	0.979	0.819	0.919

**Table 6:** Evaluation metrics and hyperparameters for ELM algorithms.

Inputs	Model	Hyperparameters (hn, af, rp)	Training		Testing	
			*MAE*	*R*	*MAE*	*R*
LMC	ELM1	160, sigmoid, 0.001	1.582	0.761	1.623	0.737
LMC, LPH	ELM2	180, sigmoid, 0.001	1.391	0.803	1.416	0.800
LMC, LPH, LT	ELM3	180, sigmoid, 0.001	1.319	0.829	1.397	0.801
T	ELM4	120, sigmoid, 0.001	1.444	0.795	1.403	0.820
T, RH	ELM5	180, sigmoid, 0.0001	1.247	0.842	1.293	0.842
T, RH, V	ELM6	180, sigmoid, 0.001	1.033	0.897	1.089	0.893
LMC, LPH, T	ELM7	180, sigmoid, 0.0001	0.992	0.901	1.109	0.872

**Table 7:** Wavelet sub-series correlations with NH_3_ data series.

Inputs	D1	D2	D3	D4	D5	D6	D7	D8	D9	D10
LMC	0.08	0.08	0.14	0.17	0.29	0.33	0.20	0.33	0.20	0.32
LPH	0.05	0.04	0.11	0.13	0.16	0.25	0.17	0.26	0.16	0.32
LT	−0.01	−0.04	−0.09	−0.16	−0.26	−0.15	−0.25	−0.24	−0.09	0.31
T	−0.03	−0.06	−0.18	−0.24	−0.30	−0.35	−0.26	−0.32	−0.22	−0.32
RH	0.03	0.05	0.10	0.11	0.11	0.24	−0.01	0.11	0.22	0.30
V	0.01	−0.02	−0.16	−0.20	−0.29	−0.18	−0.12	−0.12	0.23	0.30

**Table 8:** Comparison of correlation coefficients for inputs and NH_3_ with and without WT.

Inputs	Without WT	The New Series	With WT
LMC	0.720	D1 + D2 + D3 + D4 + D5 + D6 + D7 + D8 + D9 + D10	0.720
LPH	0.547	D3 + D4 + D5 + D6 + D7 + D8 + D9 + D10	0.551
LT	−0.398	D3 + D4 + D5 + D6 + D7 + D8 + D9	−0.499
T	0.754	D2 + D3 + D4 + D5 + D6 + D7 + D8 + D9 + D10	0.755
RH	0.393	D3 + D4 + D5 + D6 + D8 + D9 + D10	0.463
V	−0.224	D3 + D4 + D5 + D6 + D8	−0.432

**Table 9:** Evaluation metrics for the best performing ML-WT models.

Input	Model	Training		Testing	
		*MAE*	*R*	*MAE*	*R*
T, RH, V	KNN6-WT	0.391	0.984	0.716	0.948
	RF6-WT	0.224	0.992	0.548	0.976
	ELM6-WT	1.018	0.901	1.062	0.908

## Data Availability

The data presented in this study are available on request from the corresponding author.
